# A dental nightmare, resolved: what a radiologist needs to know when consulted about ingestion of dental foreign body material

**DOI:** 10.1259/bjrcr.20150166

**Published:** 2016-05-05

**Authors:** Mark Guelfguat, Jason Dipoce, James Dipoce

**Affiliations:** ^1^ Department of Radiology, Jacobi Medical Center, New York, NY, USA; ^2^ Albert Einstein College of Medicine, New York, NY, USA; ^3^ Department of Radiology, Hadassah Medical Center, Jerusalem, Israel; ^4^ Department of Radiology, Staten Island University Hospital, New York, NY, USA

## Abstract

Ingestion of dental foreign bodies, while relatively rare, may cause serious, and occasionally fatal, injuries to the airways and gastrointestinal tract. Numerous case reports are available describing the clinical course of such ingestions. The aim of this paper is to develop concise, practical recommendations to aid radiologists in providing clinically relevant diagnostic information, thereby accelerating detection and management of acute ingestion of dental material.

## Summary

Ingestion of foreign bodies is common. In the USA, 127,000 cases were reported in 1 year alone.^1^ Specific complications related to foreign body ingestion depend on the object’s size, how sharp it is and its ability to negotiate the narrow anatomical or pathological sites. Complications occur more commonly in adults compared with children.^2^ Major complications related to the foreign body ingestion are related to obstruction and perforation. Perforation is especially dangerous in the oesophagus, owing to rapid spread of inflammation along the tissue planes.^3^ Ingestion of foreign dental material follows these general principles.

Clinical presentation of foreign body ingestion has a diverse clinical appearance based on the patient’s age, location in the gastrointestinal (GI) tract, the object’s mechanical and chemical properties, the time elapsed after the ingestion and the presence of organ damage. While in paediatric patients the classic findings include excessive salivation and dysphagia, in adults, presentation can be occult or atypical, for example, mimicking sigmoid diverticulitis.^4,5^ Therefore, radiologists and clinicians alike should be on alert for the possible presence of foreign bodies in the GI and respiratory systems.

Types of ingested foreign bodies vary among the age groups. Ingestion of coins is more common among children, while bones are typically seen in the elderly, especially those who are edentulous.^2,6^ While oesophageal impaction is most common by chicken and fish in Asia, in North America, it is secondary to meat bolus.^7^

Radiographs remain the mainstay of initial foreign body detection, especially if the object is related to dental material.^6^ When swallowing of a foreign body is suspected, anteroposterior (AP) and lateral neck, AP and lateral chest, and abdominal radiographs should be obtained to perform a complete examination and search for the presence of an ingested foreign body. Although radiographs are not sensitive for detecting radiolucent substances, most dental materials are radiopaque and are thus likely to be visible radiographically. Radiographs can provide initial assessment information regarding location, size, shape and number of radiopaque foreign bodies as well as help in determining the general evidence of obstruction or perforation. Chest radiography is very helpful in demonstrating and evaluating radiopaque oesophageal foreign bodies; moreover, the lateral chest radiograph is important to distinguish between ingested foreign bodies located in the thoracic oesophagus and inhaled foreign bodies located within the trachea.^8^ Although a CT scan can be also used to localize a foreign body, it is more useful in defining the precise extent of the injury to the involved organs (*i.e.* wall erosion or perforation) and the damage to the surrounding tissues (*i.e.* pneumomediastinum, pleural effusion, ascites, pneumoperitoneum, etc.).^9^ The use of oral contrast material has been hotly debated in the literature and strong arguments have been made in support and against the use of contrast.^10–13^

Endoscopic interventions have been successful in foreign body management, with need for surgical removal in a minority of cases (1.4–16%).^9,14^ Various guidelines and algorithms have been developed over the past decades in attempts to optimize the detection and retrieval of foreign bodies, as well as to minimize complications.^4,5,9,15,16^ Regarding the radiologist, these guidelines emphasize a need for accurate reporting of the object size, potential for local damage owing to the geometrical shape or chemical composition of the foreign object, its location and migration in the human body, and observation of complications.

## Small blunt foreign body

### Dental bridge

**Figure 1.**
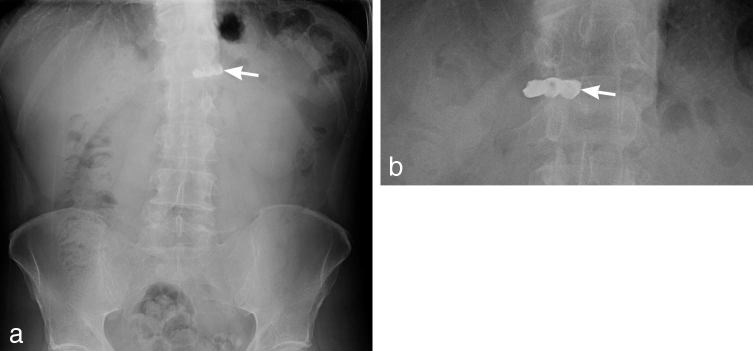
Abdominal anteroposterior radiographs (a, b) demonstrating minimal change in position of a three-unit bridge (white arrows) located in the area of the gastric antrum. The bridge is composed of three crown components attached together.

A middle-aged person swallowed a three-unit bridge (Figure 1). Intragastric location was confirmed with serial radiographs. The foreign body was removed via endoscopy, and the patient had an uneventful recovery.

### Discussion

Bridges are usually composed of porcelain fused to metal. Radiopaque metallic components are easily identified radiographically. An “egg shell” appearance of the middle crown (Figure 1a) is owing to a peripheral metal shell.^17^ Radiographic differentiation from loose teeth is made by an absence of roots. Attachments of the components help in differentiating the bridge from multiple individual crowns.

For the purposes of medical management, based on its shape and size, the bridge can be classified as a “small blunt” foreign body. Once a foreign body has reached the stomach, spontaneous evacuation is the likely outcome.^18^ Intra-gastric short blunt objects can be followed weekly with radiographs to monitor progression through the GI tract. Endoscopic removal is necessary only if the foreign body remains in the stomach after 3–4 weeks. Cases with objects remaining distal to the duodenum in the same location for longer than 1 week and patients with clinical concern for peritonitis should undergo surgical evaluation.^9^

### Learning points

1. Small blunt dental objects that have passed into the stomach can be observed with serial radiographs.
2. A failure to progress beyond the stomach after 3–4 weeks requires endoscopic retrieval.
3. Arrest of an object’s migration in the bowel is concerning for bowel obstruction or perforation. In such a case, surgical evaluation is warranted.
4. Upper digestive tract water-soluble contrast (*e.g.* gastrografin) swallow examination may be helpful for detecting, if necessary, the correct location of a small blunt ingested foreign body.

## Long foreign body

### Toothbrush

**Figure 2.**
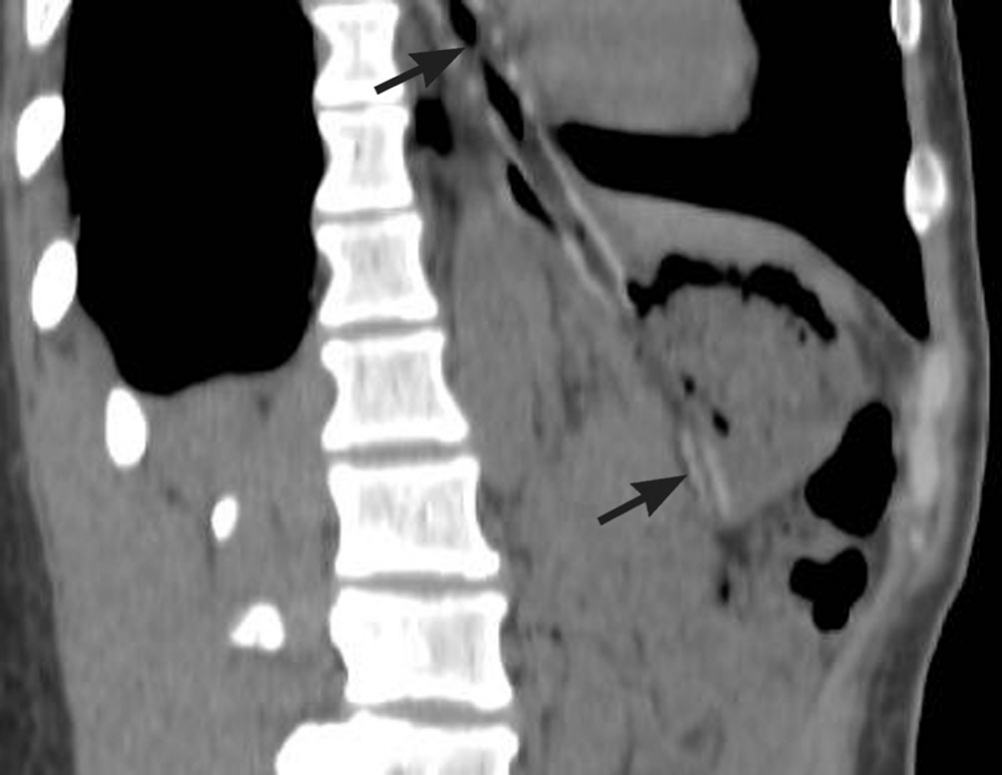
Thick maximum intensity projection CT image demonstrates a toothbrush (arrows) crossing the gastroesophageal junction. This long object (at least 17 cm) failed to descend into the stomach. As the patient feared endoscopy, which would normally be the initial approach for this foreign body removal, the toothbrush was removed surgically.

A middle-aged male with a history of mental illness was admitted multiple times over the course of 10 years for multiple episodes of toothbrush ingestions. During one of the admissions, a toothbrush failed to pass the gastroesophageal junction and was unable to descend into the stomach (Figure 2). As the entire foreign body was made of plastic, it was not apparent on radiographs. As the patient feared endoscopy, which would normally be the initial approach for this foreign body removal, the toothbrush was removed surgically.

**Figure 3.**
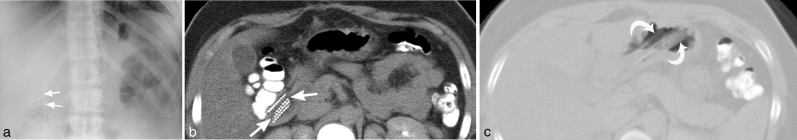
The same patient was imaged during another occurrence of toothbrush ingestion. A cropped abdominal anteroposterior radiograph (a) shows linear opacities in the right upper quadrant (arrows). A corresponding axial CT image in soft tissue window (b) reveals radiopaque bristles of stacked swallowed toothbrushes (arrows). A more cephalad CT image in a lung window (c) identifies the plastic handles that are not visible on the radiograph (curved arrows).

During another occurrence of toothbrush ingestion, the patient was found to have multiple toothbrushes in the gastric lumen (Figure 3). Again, as per the patient’s request, the toothbrushes were removed surgically.

### Discussion

In adults, foreign bodies that are longer than 6 cm and wider than 2.5 cm and are located in the stomach or proximal duodenum should be removed endoscopically.^9^ This is owing to the high risk of intra-gastric stagnation and intestinal perforation, even when the pylorus has been traversed.^18^ Segments of the GI tract with acute angulations are especially prone to perforation, with reported perforation rates of up to 73% in the ileocecal valve and appendiceal area.^5^ Thus, in addition to estimating the location of an object in the GI tract, a radiologist should report all the three dimensions of the ingested foreign body.

While radiographs were only able to identify the radiopaque bristles, CT in lung window also allowed visualization of the radiolucent plastic handles (Figure 3c). Most plastic materials, particularly if free from radiopaque additives, are usually not visible radiographically, including acrylic dental substances and toothbrushes.^19,20^ If necessary, localization of the object and estimation of size can be carried out with a CT scan. A review of the CT images in the lung and/or bone windows, in addition to the standard soft tissue window, can enhance foreign body visualization.^21^

Foreign body ingestion has a known association with history of mental illness. Although pertinent for image interpretation, this history may not be available at the time of radiological examination, and therefore, a high index of suspicion for the presence of foreign bodies is required from the radiologist.

### Learning points

1. Long objects, such as toothbrushes, should undergo urgent endoscopic removal owing to failure to progress beyond the stomach and risk of duodenal perforation while negotiating the duodenal curvature, even if it has passed beyond the pylorus.
2. Reporting of object size in three planes by the radiologist is necessary for management planning.
3. As plastic (acrylic) materials are radiolucent, non-contributory radiographic evaluation after the ingestion of plastic should be followed by a CT scan, which allows improved foreign body localization and detection of complications.

## Sharp foreign body

### Crown with a post

**Figure 4.**
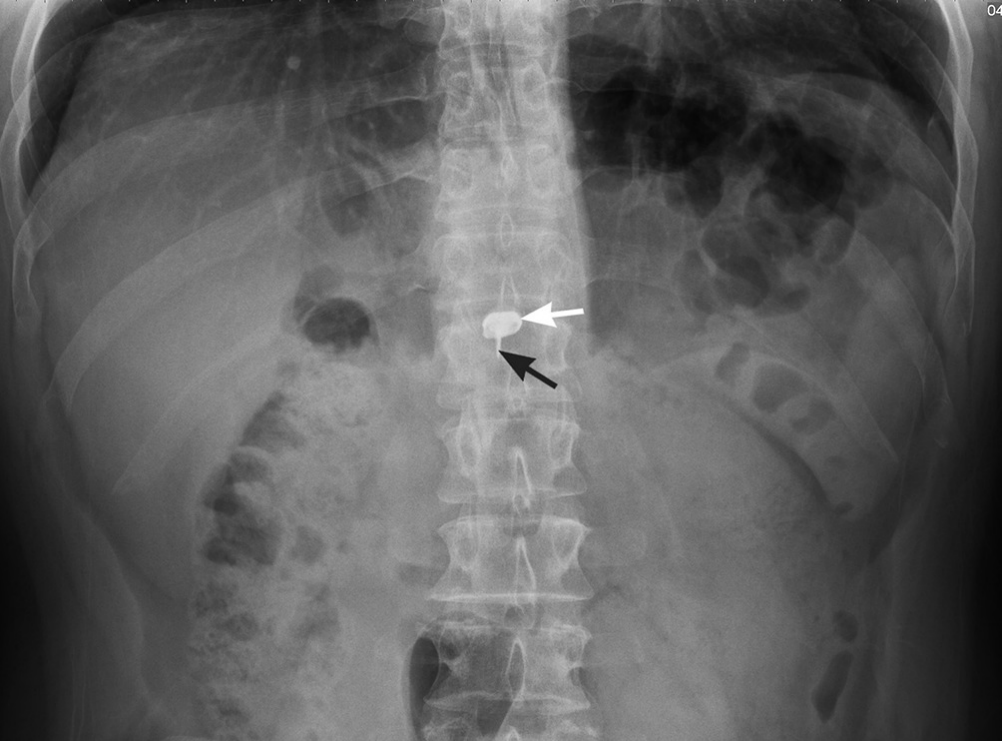
Supine abdominal anteroposterior radiograph shows a single crown (white arrow) with a post (black arrow) in the gastric antrum.

A 51-year-old male arrived at the emergency department 1 h after accidentally swallowing a crown. The initial clinical concern for oesophageal location of the ingested object was clarified after initial imaging. Radiographs determined the presence of a crown with a post in the gastric lumen (Figure 4). Subsequent to endoscopic removal, the patient was discharged in a stable condition.

### Dental drill bit

**Figure 5.**
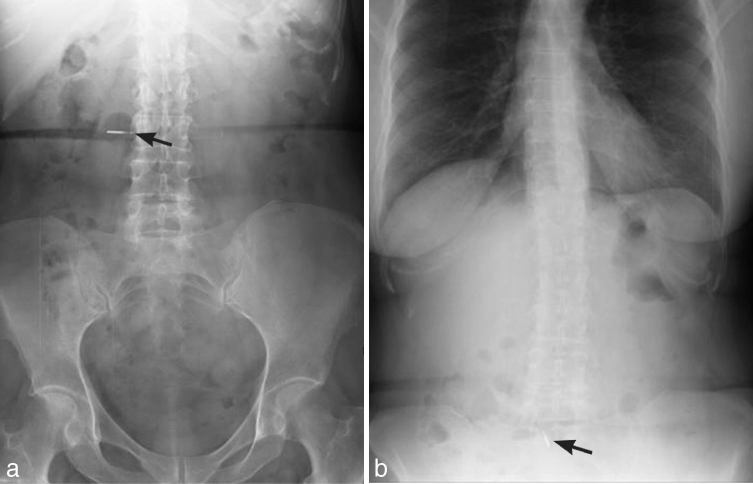
Supine abdominal anteroposterior radiographs. At presentation (a), a linear opaque right upper quadrant object, a dental bit (arrow), was presumed to be within the pre-pyloric region of the stomach or in the first portion of the duodenum. A follow-up radiograph performed on the same date (b) demonstrated migration to the pelvis.

A middle-aged female swallowed a dental bit during a dental visit. An initial abdominal radiograph revealed a linear radiopaque object in the right upper quadrant (Figure 5). The object was noted to migrate to the pelvis a few hours later on a follow-up radiograph acquired on the same day. This represented progression of the foreign body through the bowel. The patient remained asymptomatic and the bit was followed until elimination.

### Swallowed tooth

**Figure 6.**
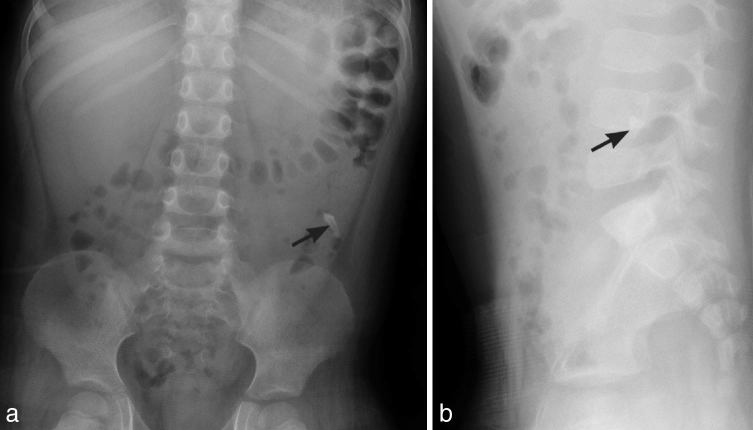
Supine anteroposterior (a) and coned-down lateral (b) abdominal radiographs. A tooth (arrows in a, b), most likely a maxillary canine, is located in the descending colon. There is a normal bowel gas pattern.

A 4-year-old male was brought to an urban emergency department with a history of possible swallowing or aspiration of a tooth after dental extraction. The patient had a benign clinical examination. Radiographs of the chest and abdomen were requested. The chest radiograph was unremarkable. The abdominal radiographs (Figure 6) showed the presence of a tooth in the descending colon and no evidence of bowel perforation. The patient was lost to follow-up. Uneventful passage of a foreign body of this size in the distal large bowel can be confirmed with stool examination and, if necessary, radiographic follow-up.

### Discussion

The fact that the ingested foreign body represents a dental bur is obvious from the provided history. If differentiation from another ingested radiopaque object is necessary (such a coin in profile), biplane radiographs can be obtained. Dental crowns, posts and drill bits are radiopaque, which facilitates radiographic identification.^17^

An ingested tooth can be easily visualized on a background of soft tissues. It may become obscured by the overlying skeleton (Figure 6b), which necessitates the acquisition of biplane radiographs for initial evaluation of the ingested foreign bodies.^22^ Swallowing of teeth can be prevented during a procedure by use of a rubber dam and throat packs.^6^

Differential diagnosis of an ingested tooth on an abdominal radiograph includes a mature teratoma. Primary retroperitoneal teratoma is an uncommon neoplasm, most often occurring in the paediatric population. Ovarian teratomas are more common and are typically asymptomatic and discovered incidentally on radiographs owing to the presence of teeth. Unlike most ingested teeth, which migrate along the length of the bowel, teeth in a teratoma remain stationary on serial radiographs. On cross-sectional imaging, teratomas appear as solid or multiloculated circumscribed masses that may contain a fatty component. Presence of a fat–fluid level is more common for ovarian teratomas than for a lesion arising at other sites.^23^

The most common location of a foreign body at the initial presentation is in the stomach (58.1%), followed by the small intestine (32.7%).^24^ Although the overall rate of perforation owing to foreign bodies is in the range of 1–7%, the incidence increases to 15–35% when sharp or pointed objects are considered.^5^ Sharp or pointed objects that have passed the oesophagus require urgent removal, if located within endoscopic reach.^18^ Once beyond the duodenum, daily radiographs are required to monitor safe passage owing to risk for perforation at the ileocecal valve.^9,18^ If, in such a case, a sharp foreign body does not progress for 3 days, surgical removal should be considered.^25^

### Learning points

1. Sharp or pointed dental objects (*e.g.* drill bits, extracted teeth) require immediate radiographic evaluation to define the location of the object. AP and lateral views are necessary to avoid obscuration by bony overlap.
2. Sharp or pointed foreign bodies that have passed into the stomach or proximal duodenum should be removed endoscopically.
3. Otherwise, safe elimination should be documented with serial radiographs. Failure to progress after 3 days requires surgical evaluation.

## Foreign body lodging at the pharynx

### Mandibular removable partial denture

**Figure 7.**
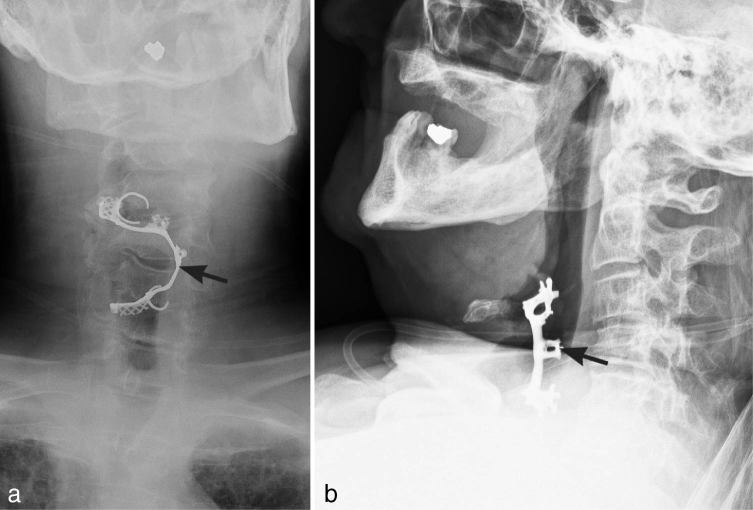
Anteroposterior (a) and lateral (b) neck soft tissue radiographs show a denture (arrows) in the hypopharynx. Mild distension of the pharynx with air, more apparent on the lateral view (b), is likely secondary to mass effect of the foreign body on the larynx.

A 70-year-old male was seen in the emergency department with complaints of cough and difficulty breathing. He reported the onset of symptoms after swallowing a denture. The denture was lodged in the hypopharynx and discovered on soft tissue neck radiographs (Figure 7). The shape of the metal frame helped in determining that the object was a mandibular removable partial denture. Emergent laryngoscopy discovered that the denture was lodged at the base of the tongue with extension into the supraglottis, immediately above the vocal cords, causing airway obstruction. The hook component was imbedded in the vallecula. Uneventful removal was conducted. Owing to observation of a small epiglottic erosion by the denture and aspiration of secretions, the patient was intubated and remained in the hospital for observation for 4 days. Serial chest radiographs obtained during the hospital stay revealed no acute disease. The patient had an uneventful recovery and was discharged home in a stable condition.

### Discussion

Although dentures comprise only 5–18% cases of foreign bodies impacted in the oesophagus, the majority of foreign body complications were reported after ingestion of dentures (80%) and bone fragments (42%).^2,7,26^ The complex shape, size (approximately 4 cm wide) and serrated contour of a dental prosthesis render it a perfect object to become impacted in the GI tract, causing severe complications.^7,27^

Oral tactile sensation diminishes with age, leading to the declining ability to control bone fragments and loose dentures, and eventually to their swallowing.^6^ Age of 50 years and above was also found to be an independent factor in complications after foreign body ingestion with oesophageal impaction.^28^ As early diagnosis is essential to avoid complications of oesophageal oedema and perforation, a high index of suspicion should be maintained when evaluating the aging population.

While most foreign bodies lodge in the oropharynx (64.5%), dentures are most commonly found in the cervical portion of the oesophagus (63%).^7,28^ The presence of a metal framework helps in identifying a partial denture. A complete denture is made entirely of plastic and would not be visible radiographically.^7^ Prevertebral soft tissue swelling has been identified in 49% of neck soft tissue radiographs after swallowing a denture. This finding rises to 79% in cases of dentures impacted in the cervical oesophagus.^7^

Medical management of a foreign body impacted in the oesophagus has been shown to be ineffective.^29^ Ingested sharp objects such as dentures, which are likely to perforate the oesophagus, require emergent endoscopic retrieval.^9^ Other retained or impacted oesophageal foreign bodies require prompt endoscopic removal within 24 h after ingestion to avoid oesophageal perforation. Further delay may lead to oesophageal ulceration.^13^ Oesophageal location of a foreign body identified radiologically should be reported with urgency.

### Learning points

1. Special considerations apply to denture ingestions, as they are known to carry a high rate of complications.
2. Since foreign body impaction in the pharynx and oesophagus is especially prone to perforation and deep soft tissue infection, a radiologist is required to immediately communicate the object’s location and conduct a search for complications.
3. A denture or any other sharp or pointed object lodged in the pharynx or oesophagus requires emergent endoscopic removal.

## Oesophageal foreign body causing perforation

### Maxillary complete denture

**Figure 8.**
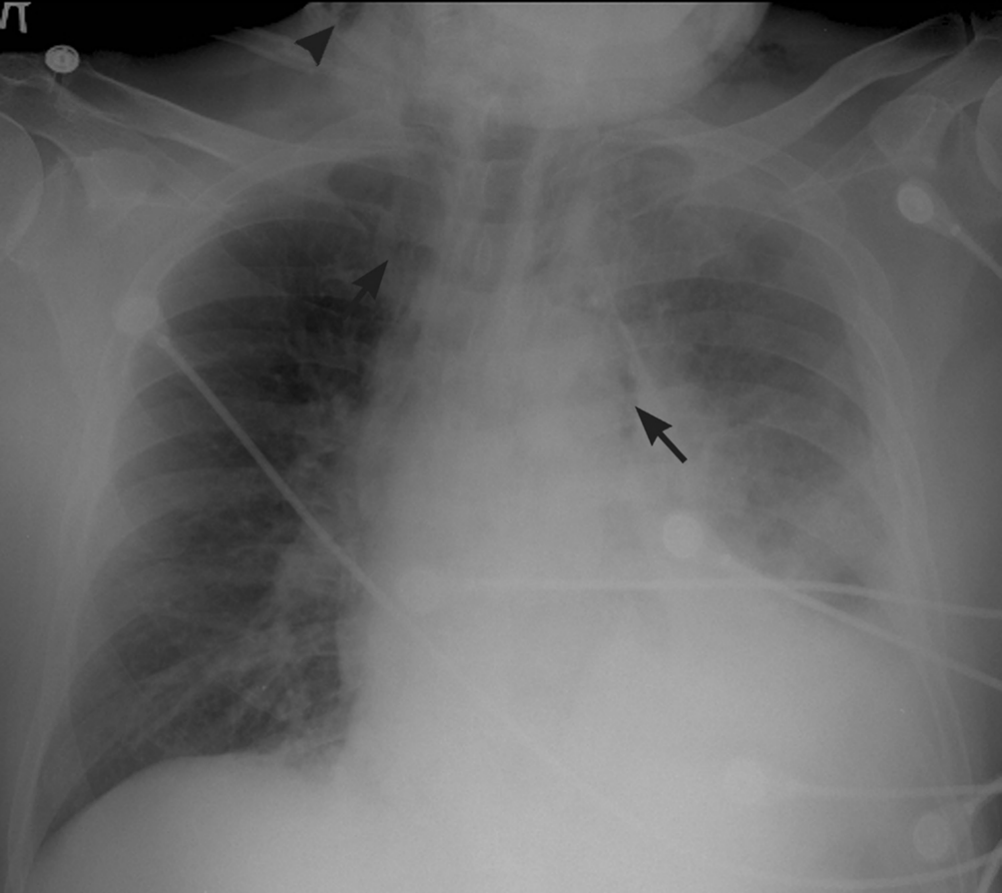
Portable anteroposterior chest radiograph shows pneumomediastinum (arrows) and soft tissue emphysema at the base of the neck (arrowhead). A denture lodged in the oesophagus is obscured by the overlying soft tissues and technique.

**Figure 9.**
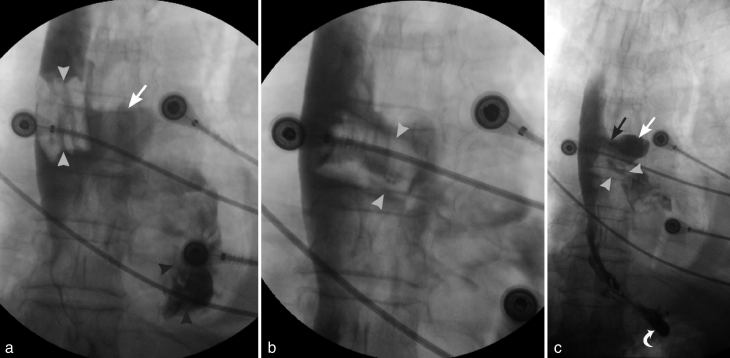
Multiple spot images obtained during fluoroscopic oesophagography. An early image (a) reveals a denture (white arrowheads) within the oesophageal lumen outlined by the oral contrast. The extraluminal oral contrast in the mediastinum (straight white arrow) and the left pleural space (black arrowheads) is noteworthy. During the course of the study (b), the denture (white arrowheads) turned horizontally and began to protrude through the oesophageal rent. One of the final images (c) demonstrates further extraluminal migration of the denture (white arrowheads). Oral contrast outlines the cephalad margin of the midthoracic oesophageal tear (black arrow) communicating with the mediastinal contrast collection (straight white arrow). Oral contrast transit into the stomach is evident (curved white arrow).

An elderly male presented to an emergency department with chest pain after swallowing a maxillary complete denture. Chest radiograph findings of pneumomediastinum and cervical soft tissue emphysema were concerning for oesophageal perforation by the ingested denture (Figure 8). Subsequent fluoroscopic oesophagography with water-soluble contrast revealed thoracic oesophageal tear with extraluminal contrast accumulation in the mediastinum and left pleural space (Figure 9). During the course of the study, the denture, initially aligned longitudinally along the oesophageal lumen, rotated horizontally and began to extend into the mediastinum through the oesophageal rent. Emergent surgical oesophageal repair with removal of the extruded foreign body was performed. Despite extensive thoracic injury, the patient had a successful recovery.

### Discussion

Foreign bodies typically impact in the oesophageal lumen at the levels of physiologic narrowing or oesophageal disease. Prolonged stasis leads to oesophageal perforation and even injury to the nearby organs. Mortality from oesophageal perforation is close to 20%. Sharp foreign bodies, most commonly dental prostheses and bones, are the typical causes of oesophageal perforation.^30^ Sharp-edged objects, such as dentures, can rapidly erode through the oesophageal wall and result in cervical and mediastinal infections, damage to the major vessels and significant haemorrhage.^31^

Chest radiographs can show indirect findings of oesophageal perforation, such as pleural effusions, pneumoperitoneum, pneumothorax, hydropneumothorax and pneumomediastinum.^7,30^ The detection of prevertebral soft tissue gas on lateral cervical radiographs is an early radiographic sign of oesophageal perforation.^3^ Radiographs in both AP and lateral projections are necessary, since soft tissue gas and foreign bodies may be obscured on a single view.^22^

Fluoroscopic oesophagography with water-soluble agents has a sensitivity of 50% for detection of cervical oesophageal perforation and 75–80% for detection of thoracic oesophageal perforation. It may help in localizing the site of perforation, estimating the size of the tear, and determining the mediastinal or pleural extent of the leak.^11,30^ As barium is more sensitive than oral water-soluble contrast for detecting small perforations, some advocate barium re-evaluation after a normal-appearing water soluble oesophagography examination.^32^ Of note, endoscopic literature strongly advises against oral contrast use, as it may limit subsequent endoscopic visualization.^9,13^

CT scan is the preferred imaging study for evaluation of oesophageal perforation. It allows detection of the offending foreign body and the visualization of oesophageal wall thickening at the perforation site. Most importantly, a CT scan can demonstrate the extent of mediastinal and pleural involvement, as well as visualize fistulas and abscesses, thus aiding surgical planning and guiding the operative approach.^10,11,22^ Communicating to the clinicians the extent of mediastinal disease evident on a CT scan is of particular importance, as endoscopic management may be attempted in the absence of perioesophageal abscess and if the duration of the foreign body impaction is less than 24 h.^13^

Oral contrast enhances CT differentiation of a contained versus non-contained leak, and estimation of oesophageal tear size and location. Nevertheless, no clear guidelines exist regarding utilization of oral contrast in CT scan, as this issue remains the subject of debate.^10^

Differential diagnosis of oesophageal perforation includes epiphrenic diverticulum, Zenker’s diverticulum and circumferential submucosal dissection of the oesophagus. An epiphrenic diverticulum can be distinguished by the characteristic origin from the lower thoracic oesophagus, wide-mouth communication with the oesophageal lumen and lack of surrounding inflammatory changes in the mediastinum.^33^ Zenker’s diverticulum is typically located at the C5–6 level. Rounded contours help to tell it apart from the irregular shape of an extraluminal fluid leak.^34,35^ Circumferential submucosal dissection of the oesophagus is a result of detachment of the oesophageal mucosal and submucosal layers. Imaging helps in visualizing the formation of a false lumen communicating with the true lumen. A lack of secondary mediastinal signs of perforation confirms the diagnosis.^36^

### Learning points

AP and lateral radiographs of the neck and chest are used in initial screening of an oesophageal perforation. A CT scan is a sensitive and specific modality for evaluation of patients with suspected pharyngoesophageal foreign bodies. It is particularly useful in detecting impacted foreign bodies and determining the extent of complications. Reporting of this information to the clinicians has a direct influence on the pre-procedural planning.

## Conclusions

Ingestion of a foreign body is commonly encountered in radiological practice. Nevertheless, the significance of swallowed foreign bodies should not be underestimated. The majority of small dental objects, such as teeth and dental bits will uneventfully pass through the GI tract and can be followed with serial radiographs. Some cases, such as denture ingestion, will require early intervention and advanced imaging. When lodging in the pharynx or oesophagus occurs, a directed imaging investigation should be conducted to evaluate for the presence of predisposing risk factors, underlying oesophageal pathology and potential complications.

Assessment of an ingested dental foreign body should include evaluation of the object type and location, as well as considerations that are likely to predict the likelihood of a perforation, such as relationship relative to the GI sites of anatomic narrowing (such as sphincters and angulations), size and shape of the foreign body, age of the patient and duration of the object lodging at that specific location. The ability to determine these factors places the radiologist in a critical position, providing the clinician with crucial patient care information. Maintaining the proper perspective of management issues also aids in producing a meaningful imaging report.

## Consent

Informed consent from the patient/guardian/next of kin for the case to be published (including images, case history and data) could not be obtained. Exhaustive efforts were made to contact the patient/guardian/next of kin over a 6 month period but proved unsuccessful. Patient data has been anonymized to protect patient identity.
